# Cationic Polymer Brush-Modified Carbon Nanotube-Meditated eRNA LINC02569 Silencing Attenuates Nucleus Pulposus Degeneration by Blocking NF-κB Signaling Pathway and Alleviate Cell Senescence

**DOI:** 10.3389/fcell.2021.837777

**Published:** 2022-01-17

**Authors:** Yulin Huang, Jiaming Yang, Xizhe Liu, Xiaoshuai Wang, Kai Zhu, Zemin Ling, Baozhu Zeng, Ningning Chen, Shaoyu Liu, Fuxin Wei

**Affiliations:** ^1^ Department of Orthopedics Surgery, The Seventh Affiliated Hospital of Sun Yat-sen University, Shenzhen, China; ^2^ Guangdong Provincial Key Laboratory of Orthopaedics and Traumatology/Orthopaedic Research Institute, Department of Spine Surgery, The First Affiliated Hospital of Sun Yat-sen University, Guangzhou, China; ^3^ Clinical Research Centre, Zhujiang Hospital, Southern Medical University, Guangzhou, China; ^4^ Orthopaedic Section II, Affiliated Dongguan Hospital, Southern Medical University, Dongguan, China; ^5^ Guangdong Provincial Key Laboratory of Orthopedics and Traumatology, Department of Spinal Surgery, The First Affiliated Hospital of Sun Yat-sen University, Guangzhou, China

**Keywords:** Enhancer RNA, intervertebral disc degeneration, carbon nanotube, inflammation, senescence

## Abstract

Enhancer RNAs (eRNAs) are noncoding RNAs that synthesized at active enhancers. eRNAs have important regulatory characteristics and appear to be significant for maintenance of cell identity and information processing. Series of functional eRNAs have been identified as potential therapeutic targets for multiple diseases. Nevertheless, the role of eRNAs on intervertebral disc degeneration (IDD) is still unknown yet. Herein, we utilized the nucleus pulposus samples of patients and identified a key eRNA (LINC02569) with the Arraystar eRNA Microarray. LINC02569 mostly locates in nucleus and plays an important role in the progress of IDD by activating nuclear factor kappa-B (NF-κB) signaling pathway. We used a cationic polymer brush coated carbon nanotube (oCNT-pb)-based siRNA delivery platform that we previously designed, to transport LINC02569 siRNA (si-02569) to nucleus pulposus cells. The siRNA loaded oCNT-pb accumulated in nucleus pulposus cells with lower toxicity and higher transfection efficiency, compared with the traditional siRNA delivery system. Moreover, the results showed that the delivery of si-02569 significantly alleviated the inflammatory response in the nucleus pulposus cells via inhibiting P65 phosphorylation and preventing its transfer into the nucleus, and meanwhile alleviated cell senescence by decreasing the expression of P21. Altogether, our results highlight that eRNA (LINC02569) plays important role in the progression of IDD and could be a potential therapeutic target for alleviation of IDD.

## Introduction

Low back pain (LBP) is one of the leading causes of disability worldwide, and the disability that caused by LBP increased by 54% between 1990 and 2015 globally (Hartvigsen et al., 2018). It hase been reported that the incidence of LBP is closely correlated with IDD (Smith et al., 2011), which is the basis of a series of lumbar spine diseases (e.g., lumbar disc herniation). IDD is a multifactorial process characterized by cellular and biomechanical changes that lead to loss of extracellular matrix (ECM) proteoglycans, increased fibrillation and reduction of water content (Antoniou et al., 1996). It is established that progressive inflammatory response plays critical role in the development of disc degeneration and LBP (Khan et al., 2017; Zhang et al., 2019). The inflammatory-associated factors tumor necrosis factor-α (TNF-α), interleukin-1β (IL-1β) and interleukin-6 (IL-6) are widely reported to be associated with IDD (Le Maitre et al., 2005; Le Maitre et al., 2007). Both the *in vitro* and *in vivo* studies have also demonstrated that inflammatory stimulation was able to trigger the degeneration of nucleus pulposus cells (NPCs) (Srivastava et al., 2017; Hernandez et al., 2020), (Rajan et al., 2013). Thus, attenuation of the inflammatory response in the intervertebral discs has long been considered as a specific therapeutic molecular target for treatment of IDD (Chou et al., 2020; Shao et al., 2020; Lyu et al., 2021).

Evidence has indicated that noncoding RNAs, including long non-coding RNAs (lncRNAs) and microRNAs (miRNAs), may play an important role in the generation of IDD (Tang et al., 2020; Zhou et al., 2021; Zhu et al., 2021). The noncoding RNAs, synthesized at active enhancers, are called eRNAs (Schaukowitch et al., 2014). These eRNAs have important regulatory characteristic, including cell/tissue specificity, and appear to be significant for maintenance of cell identity and information processing (Li et al., 2013; Mousavi et al., 2013; Sigova et al., 2015; Tsai et al., 2018). Currently, series of functional eRNAs have been identified as potential therapeutic targets for multiple diseases (e.g., cancer, cardiac fibrosis, leukemia) (Lai et al., 2013; Micheletti et al., 2017; Rahnamoun et al., 2017; Catarino and Stark, 2018; Miao et al., 2018). Although the regulatory mechanisms of these eRNAs have been generally expounded, their clinical translation is significantly impeded due to few efficient delivery strategies for regulating the biological functions of eRNAs. In addition, the proportion of currently discovered functional eRNAs is still very low and their regulatory mechanisms need to be explored. Recently, a new class of lncRNAs has emerged as lnc-eRNA or elncRNA, which are encoded in enhancer regions marked by histone 3 lysine 4 monomethylation (H3K4me1) and histone 3 lysine 27 acetylation (H3K27ac) (Miao et al., 2018), and the regulatory roles of these lncRNAs, especially those in the progression of IDD, has not been explored.

In this study, we utilized the nucleus pulposus (NP) samples of the patients who underwent discectomy in our hospital and identified a key lncRNA (LINC02569) with the Arraystar Microarray, which plays an important role in the progression of IDD via activating the NF-κB signaling pathway. Based on the regulatory mechanism, a oCNT-pb-based siRNA delivery platform that we previously designed, was used to transport si-02569 to NPCs for the attenuation of IDD. After administration, the siRNA loaded nanoparticles could accumulate in the NPCs with lower toxicity and higher transfection efficiency, compared with the traditional siRNA delivery system. Moreover, the results showed that delivery of si-02569 with this oCNT-pb platform could significantly alleviate the inflammatory response in the NPCs via regulating NF-κB signaling pathway. Taken together, these results highlighted the potential for oCNT-pb-based siRNA delivery of eRNA as a therapeutic strategy for the amelioration of inflammation mediated IDD.

## Materials and Methods

### Tissues

Human lumbar disc tissues were obtained from patients who were performed NP resection due to disc herniation in our hopsital (Guangdong, China). The degree of IDD was determined by magnetic resonance imaging (MRI) scan following the Pfirrmann classification (Pfirrmann et al., 2001). Tissues of Pfirrmann I-II degree were used as normal control. This study was approved by the ethics committee of the Seventh Affiliated Hospital of Sun Yat-sen University.

### Extraction and Culture of Human Nucleus Pulposus Cells

The NP tissues were digested in 0.2% type II collagenase (9001-12-1, BioFroxx) for 4 h at 37°C. After being washed with PBS, the digested tissues were transferred to DMEM/F12 (C11330500BT, Gibco, Life technologies) containing 10% fetal bovine serum (P30-3306, PAN Biotech) and 1% penicillin/streptomycin (15140-122, Gibco, Life technologies) in the incubator at 5% CO_2_ and 37°C. When confluent, the cells were passaged after digesting with 0.25% Trypsin-EDTA (25200-072, Gibco, Life technologies). Cells after the second passage were used in the following experiments.

### Sa-β-Gal Staining

SA-β-Gal staining was conducted using Senescence β-Galactosidase Staining Kit (C0602 ,beyotime) according to the manufacturer’s instructions. In brief, after washed with PBS once, cells on plates were fixed with 4% Paraformaldehyde (BL539A, biosharp) for 15 min at room temperature. After that, cells were stained with working solution overnight in the absence of CO_2_. Images were captured using Leica DMI1 microscope and the percentages of SA-β-gal-positive cells were quantified for statistical analysis.

### RNA Isolation and qRT-PCR

Total RNA was isolated from NPCs or tissues of NP and annulus fibrosus (AF) with TRIzol (15596026, Life technologies, United States) according to the manufacturer’s instructions. The concentration of RNA was detected using a NanoDrop spectrophotometer (Thermo Scientific, United States). cDNA synthesis was performed with PrimeScript™ RT reagent Kit (RR047A, Takara) by T100 Themal Cycler (Bio-Rad, United States). qRT-PCR was then performed with PowerUp™ SYBR™ Green Master Mix (A25742, Thermo Fisher Scientific, United States) by CFX96 Real-Time System (Bio-Rad, United States) in triplicate in three independent experiments. GAPDH was used as the internal control for mRNA and lncRNA expression. The relative expression was determined by the 2^−ΔΔCt^ method. The primer sequences used in this study are shown in Supplementary Table S1.

### Western Blot Analysis

RIPA Lysis Buffer (P0013K, Beyotime) and protease and phosphatase inhibitor cocktail (P1045, Beyotime) were used to isolate whole cell protein. Protein concentration was quantified with Pierce™ BCA Protein Assay Kit (23227, Thermo Scientific). Proteins were boiled with 5 × loading buffer, then separated by SDS-PAGE on polyacrylamide gels (PG213, EpiZyme). After that, samples were transferred to PVDF membranes (IPVH00010, Millipore). After blocking with 5% non-fat milk (P0216-1500g, Beyotime) for 1 h, membranes were incubated with primary antibodies at 4°C for 12–16 h. The next day, membranes were washed with TBS/Tween20 (TBST) and incubated with secondary antibodies at room temperature for 1 h. The protein signal was visualized by ECL chemiluminescence kit (P0018AS, Beyotime). Information for primary antibodies is shown in Supplementary Table S2.

### Materials for the Synthesis of oCNT-pb

Materials used in this study was previously reported (Li et al., 2021). In brief, pristine-multi-walled-carbon-nanotubes (pCNT) were-purchased-from-Nanostructured-&-Amorphous-Materials-Inc., United States. Sulfuric acid (H_2_SO_4_, 98%) was-purchased-from-Honywell-Fluka™, Germany. Nitric-acid (HNO_3_, 65%) was-purchased-from-Acros-Organics, United States. Dopamine-hydrochloride (98%), triethylamine (Et_3_N, 99%), α-bromoisobutyryl-bromide (α-BiBB, 98%), 2-dimethylaminoethyl-methacrylate (DMAEMA, 98%), 2,2′-bipyridyl (bipy, 99%), copper (II) bromide (CuBr_2_, 99%), copper (I) chloride (CuCl, 99.995%) were-purchased-from-Sigma-Aldrich. Dopamine-hydrochloride-and CuCl-were-kept-sealed-until-use-and-purged-with-N_2_-gas-after-every-use-to-avoid-oxidation.

### Synthesis and Characterization of oCNT-pb

The synthesis of oCNT-pb, was previously reported *via* “grafting from” method (Li et al., 2021). The procedure includes the oxidation of the pristine carbon nanotubes (CNTs), the coating of polydopamine on the oxidised CNTs, the deposition of initiators and the polymerisation of cationic polymer brushes on CNTs. Briefly, pristine CNTs were oxidised with a mixed acid solution of H_2_SO_4_ and HNO_3_. Then, polydopamine was coated on carbon nanotubes through π-π interaction with spontaneous oxidative polymerisation of dopamine hydrochloride in 10 mM tris-HCl buffer (pH 8.5), followed by the anchoring of α-BiBB initiators on CNTs *via* esterification reaction. Finally, the cationic PDMAEMA brushes were functionalized on CNTs with controlled architecture *via* surface-initiated atom transfer radical polymerisation (SI-ATRP).

The physicochemical properties, which include zeta potential (for surface charge), transmission electronic microscope (TEM, for morphology), thermogravimetric analysis (TGA, for polymer brush weight composition) and attenuated total reflection-fourier transform infrared spectroscopy (ATR-FTIR, for chemical functionalization) of the obtained oCNT-pb were characterized.

### Transfection With siRNA

The transfection using oCNT-pb was performed as the following method: diluted the oCNT-pb and siRNA in Opti-MEMTM reduced serum medium (31985062, Thermo Fisher). Mixed oCNT-pb and siRNA solution and incubated for 10min at room temperature. Then add the mixed solution to the plate to make the final concentration at 50 μM for oCNT-pb and siRNA. The transfection using riboFECT™ CP (C10502-05, Robobio) was performed as the manufacturer’s instruction. The si-02569 and Fam labeled control siRNA was purchased from RiboBio Company. NPCs were seeded in 6-well plates and incubated in 2 ml of medium for 24 h. Then, the siRNA-oCNT-pb or siRNA-riboFECT complexes were added to silence the LINC02569 expression. After incubation for 6 h, cells were washed with PBS and incubated in fresh medium for another 48 h. The sequence of siRNA-LINC02569: 5′-GGT​CGT​ATC​TTT​ATC​TGG​T-3’.

### Fluorescence In Situ Hybridization

Ribo™ Fluorescent *In Situ* Hybridization kit (R11060.7, Ribobio) was used according to the manufacturer’s instruction to measure the subcellular localization of LINC02569. In brief, after treated with 4% Paraformaldehyde and Triton X-100 (A110694-0100, Sangon Biotech), the cell slide was treated with LINC02569 probe hybridization solution labeled by Ribobio. The slide was hybridized at 37°C overnight and immersed in 4X, 2X and 1X SSC buffer (ES-8216, EcoTop Bio) in order, followed by staining with mounting medium with DAPI (ab104109, Abcam). The slide was imaged using the Zeiss LSM880 confocal microscope (Leica, Germany).

### Immunofluorescence

Cells seeded on plates were treated with 4% Paraformaldehyde and Triton X-100, then blocked by goat serum (ZLI-9022, ZSGB-Bio) for 60 min at room temperature. Primary antibodies against COL2A1 (1:100) and ACAN (1:100) were applied to the incubation at 4°C overnight. Then, the slides were incubated with Alexa Fluor^®^647- labelled second antibodies (1:500) for 1 h and stained with mounting medium with DAPI. Last, slides were observed using the Zeiss LSM880 confocal microscope. ImageJ software 1.0 (Bethesda, MD, United States) was used for quantification of images.

### Chromatin Immunoprecipitation Followed by Sequencing

ChIP was performed using the SimpleChIP^®^ Plus Sonication ChIP kit (Cell Signaling Technology, Danvers, MA, United States) according to the manufacturer’s protocol. An anti-H3K27ac antibody (8173S, Cell Signaling Technology) was used. H3K27ac-ChIP DNA and the input DNA of NPCs were used for ChIP-Seq analyses (Aksomics, Shanghai, China). A region with a *p*-value (−10*log) ≥ 50 was defined as a GATA6-enriched region.

### Cell Counting Kit-8 Assay

Cell proliferation was measured using cell counting kit-8 (CCK-8) (CK04, Dojindo). Cells (1 × 10^4^/well) were seeded into a 96-well plate and incubated for 24 h. After treated with different concentration of oCNT-pb for 6 h, 10 μl CCK-8 reagent was added to each well at the time of harvest. After incubating at 37°C for 1 h, The absorbance at 450 nm was measured using the microplate reader (Synergy H1, BioTek). The data are representative of 3 independent experiments.

### Cell Apoptosis Analysis

Cells were seeded in a 12-well plate (2 × 10^5^ cells/well). After treated with oCNT-pb or riboFECT for 6 h, cells were harvested by 0.25% Trypsin-EDTA and washed with PBS three times, and then incubated with 1 μl of FITC-conjugated Annexin V (640906, Biolegend) and 0.5 μl of PI (79997, Biolegend) for 10 min at room temperature. The stained cells were detected by the flow cytometer (CytoFLEX, Beckman).

### RNA Sequencing

NPCs were transfected with or without si-02569. Cells were harvested 48 h after transfection. Cellular RNAs were extracted from cell lysates using Trizol reagent. Total RNA is enriched by oligo (dT) magnetic beads (rRNA removed). KAPA Stranded RNA-Seq Library was used for RNA-seq library preparation.

### Statistical Analysis

GraphPad Prism 8.0 (GraphPad Software, La Jolla, CA, United States) was used for statistical analysis. Data are presented as the mean ± SD. Graphs were generated with GraphPad Prism 8.0. Statistical analysis between 2 groups was performed using student’s t-test. *p < 0.05, **p < 0.01, ***p < 0.001 and ****p < 0.0001 were regarded as statistically significant.

## Results

### LINC02569 Is Highly Expressed in Degenerative Nucleus Pulposus

**FIGURE 1:**
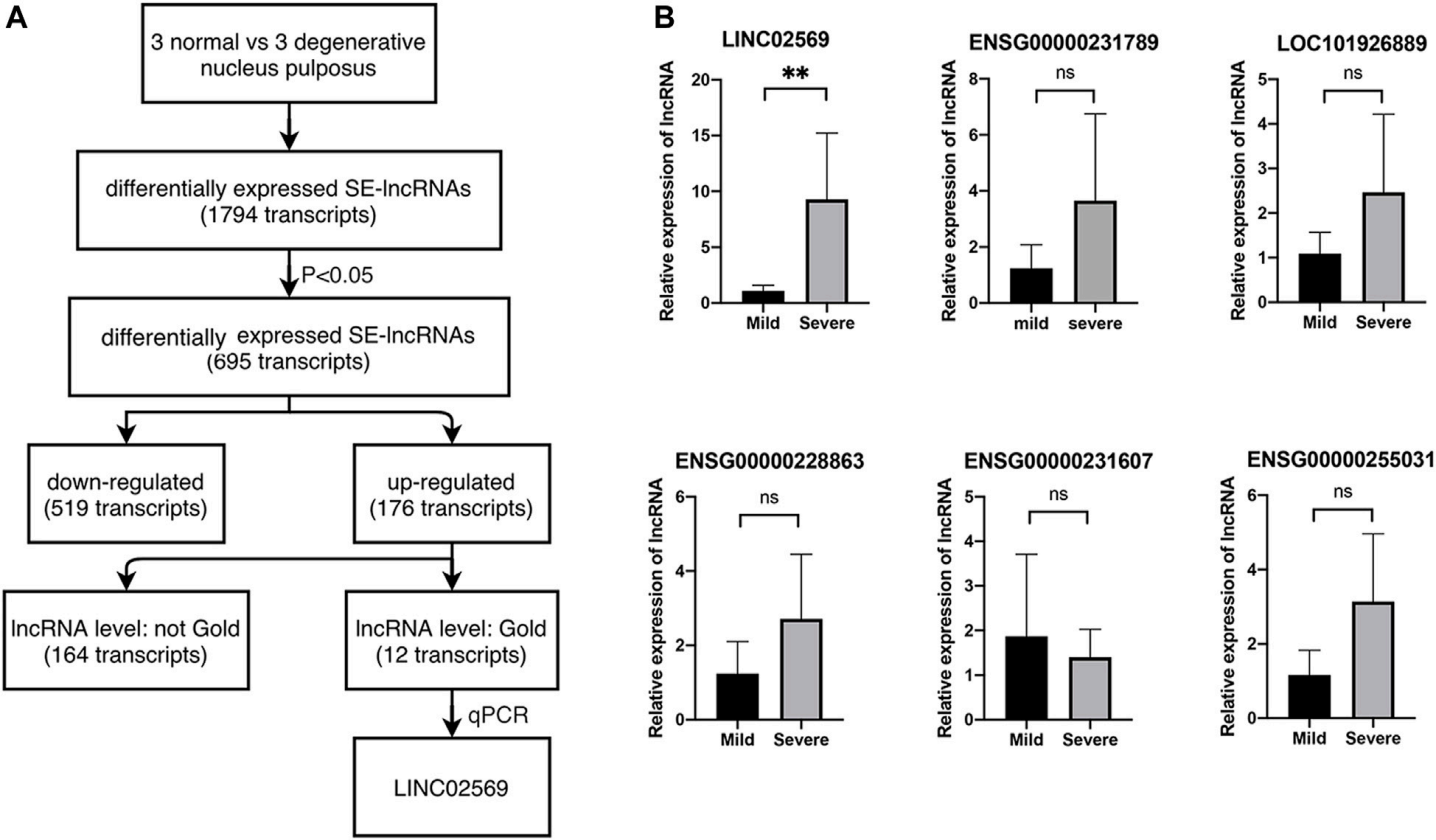
Identification of lncRNAs. **(A)** Selection strategy of lncRNAs from a genome-wide profiling between normal and degenerative NPs. **(B)** Expression of LINC02569 and other 5 lncRNAs measured by qRT-PCR in severe degenerative NPCs as compared to mild degenerative ones. Quantitative results are shown as the mean ± SD. ∗∗*p* < 0.01. ns, not statistically significant.

To investigate the role of eRNA in the progression of NP degeneration, we set out to identify eRNAs modulated in degenerative NPs compared with normal ones with Arraystar Human SE-lncRNA Microarray (Figure 1A). We first filtered 695 differentially expressed lncRNA in degenerative NPs, of which 176 lncRNA was upregulated. Then we filtered for lncRNA with gold level. As a result, 12 of them were left. Next, 6 out of the 12 were picked and expression of these transcripts was determined via qRT-PCR using RNA isolated from NP tissues. We verified that LINC02569 was significantly up-regulated in degenerative NPs (Figure 1B), supporting the notion that LINC02569 could play an important role in the progression of NP degeneration.

### LINC02569 expression Is Enriched in Nucleus Pulposus and Associated With Nucleus Pulposus Degeneration

**FIGURE 2:**
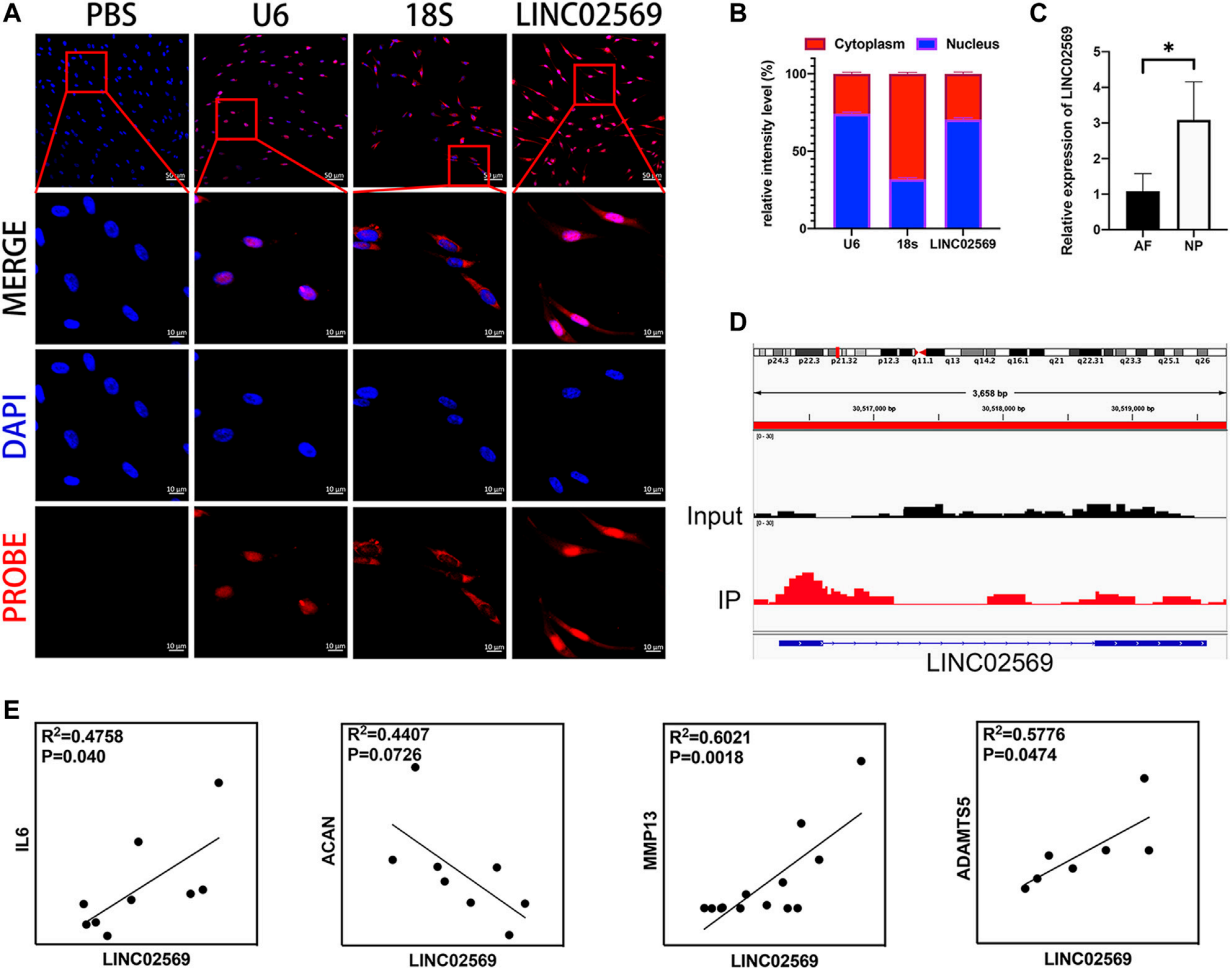
LINC02569 is eRNA and relevant to the progression of NP degeneration. **(A)** NPCs were stained with LINC02569 probe (red) and nuclear marker (DAPI, blue), while U6 and 18S was used as nucleus marker and cytoplasm marker respectively. Scale bar: 10 μm **(B)** Percentage of nucleus (blue bar) and cytoplasmic (red bar) average fluorescence intensity of U6, 18S and LINC02569 measured by ImageJ 1.0 after FISH. **(C)** qRT-PCR assay of LINC02569 expression in NP or AF. **(D)** ChIP-seq assay of H3K27ac signature of the locus encompassing LINC02569 in NPCs. **(E)** Scatter plot of qPCR assay showed correlation of expression between LINC02569 and IL6, ACAN, MMP13 and ADAMTS5. R2 and *p* values were determined by Pearson’s correlation test. Quantitative results are shown as the mean ± SD. ∗*p* < 0.05.

According to NCBI database, LINC02569 is a transcription from chromosome 6. It was found to be distributed mostly in the nucleus compartments (Figures 2A,B), indicating that it could mainly play roles in transcriptional regulatory processes. LINC02569 was more highly enriched in NP than in AF (Figure 2C, *p* < 0.05), suggesting that it could have more important functions in this intervertebral disc composition. Interestingly, despite the annotation of the SE-lncRNA microarray, we found that LINC02569 was derived from enhancer region (Figure 2D). It was more accurate to defined it as elncRNA. Since LINC02569 was enriched in degenerative NP, we therefore evaluated whether the expression of LINC02569 correlated with genes linked to NP degeneration. LINC02569 expression was highly correlated with genes relevant to inflammation and ECM degradation (Figure 2E).

### Characterization of oCNT-pb

**FIGURE 3:**
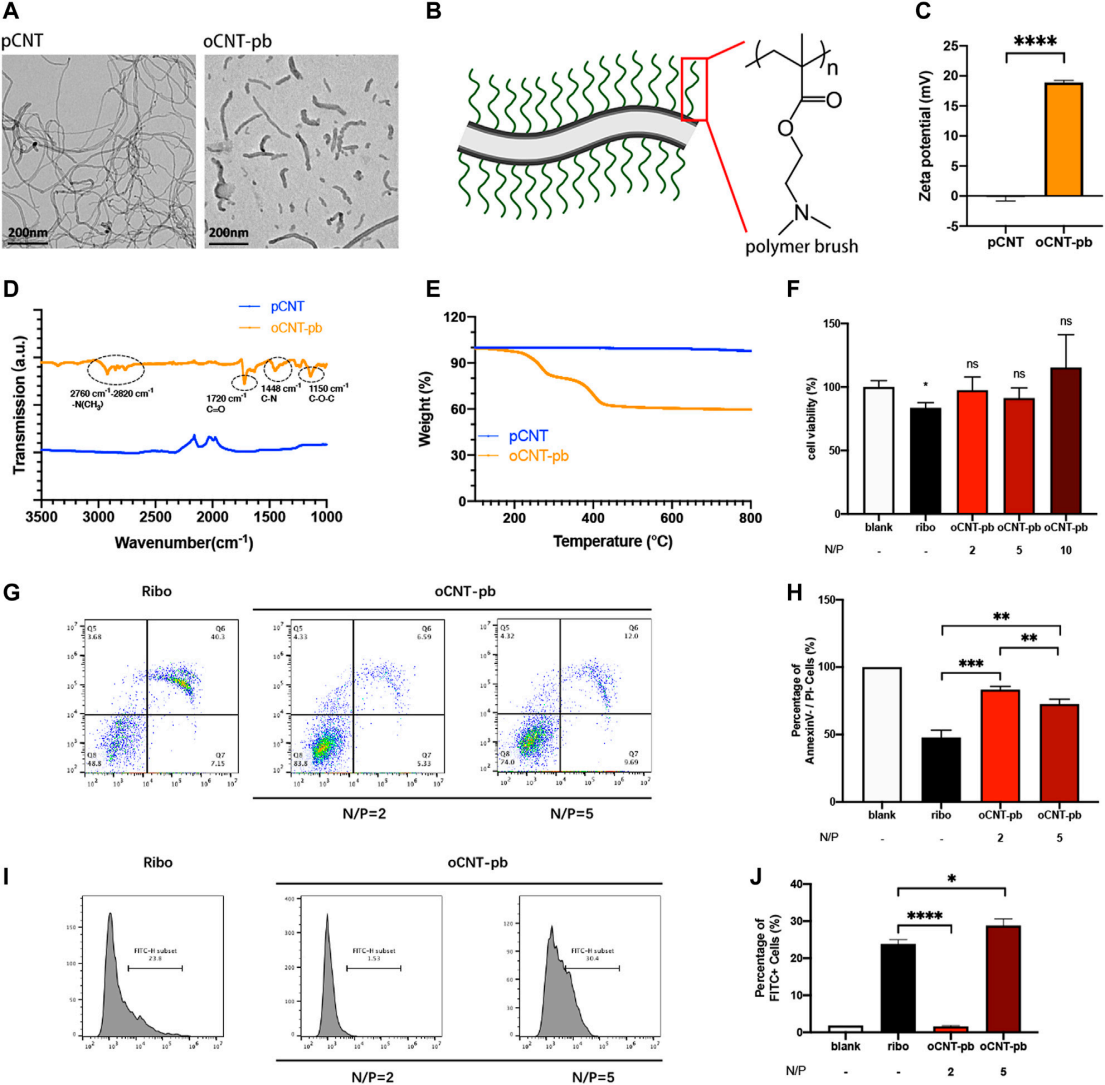
Characterization of oCNT-pb. **(A)** TEM image of pCNT and oCNT-pb. **(B)** Schematic diagram of oCNT-pb and the chemical structure of polymer brush. Scale bar: 200 nm **(C)** Zeta potential measurements of pCNT and pCNT-pb in 10 mM PBS. **(D)** FTIR of pCNT and pCNT-pb with wavenumber range from 1000 to 3500 cm^−1^. **(E)** TGA of pCNT and oCNT-pb measured from 150 to 800°C. **(F)** The cytotoxic effect of oCNT-pb at various concentrations and Ribo transfection reagent on NPCs for 6 h was determined using a CCK8 assay. PBS was used as control. **(G)** Flow cytometry profile and **(H)** percentage of apoptosis of NPCs treated with oCNT-pb at various concentrations and Ribo transfection reagent for 6 h. **(I)** Flow cytometry profile and **(J)** percentage of FITC positive cells after NPCs were transfected with Fam-labeled control siRNA by oCNT-pb at various concentrations and Ribo transfection reagent for 6 h. Quantitative results are shown as the mean ± SD (n = 3). *p < 0.05, **p < 0.01, **p < 0.001, ****p < 0.0001.

The siRNA deliver system named oCNT-pb, showed a tube-like morphology with an average size of ≈200 nm, coated with polymer brush (Figures 3A,B). The physicochemical properties, which include zeta potential (Figure 3C, for surface charge), attenuated total reflection-fourier transform infrared spectroscopy (Figure 3D, ATR-FTIR, for chemical functionalization) and thermogravimetric analysis (Figure 3E, TGA, for polymer brush weight composition) of the oCNT-pb were characterized. Before transfection, we explored the concentration of oCNT-pb. CCK8 assay showed that oCNT-pb at N/P ratio of 2, 5 and 10 did not lead to reduced viability in NPCs, while RiboFECT led to a slight reduction (Figure 3F, *p* < 0.05). Moreover, oCNT-pb at N/P ratio of 5 showed less apoptosis (Figures 3G,H) and more transfection efficiency (Figures 3I,J). So oCNT-pb at N/P ratio of 5 was used in the following experiment.

### Knockdown of LINC02569 Attenuated Nucleus Pulposus Cells Degeneration and Alleviated Senescence

**FIGURE 4:**
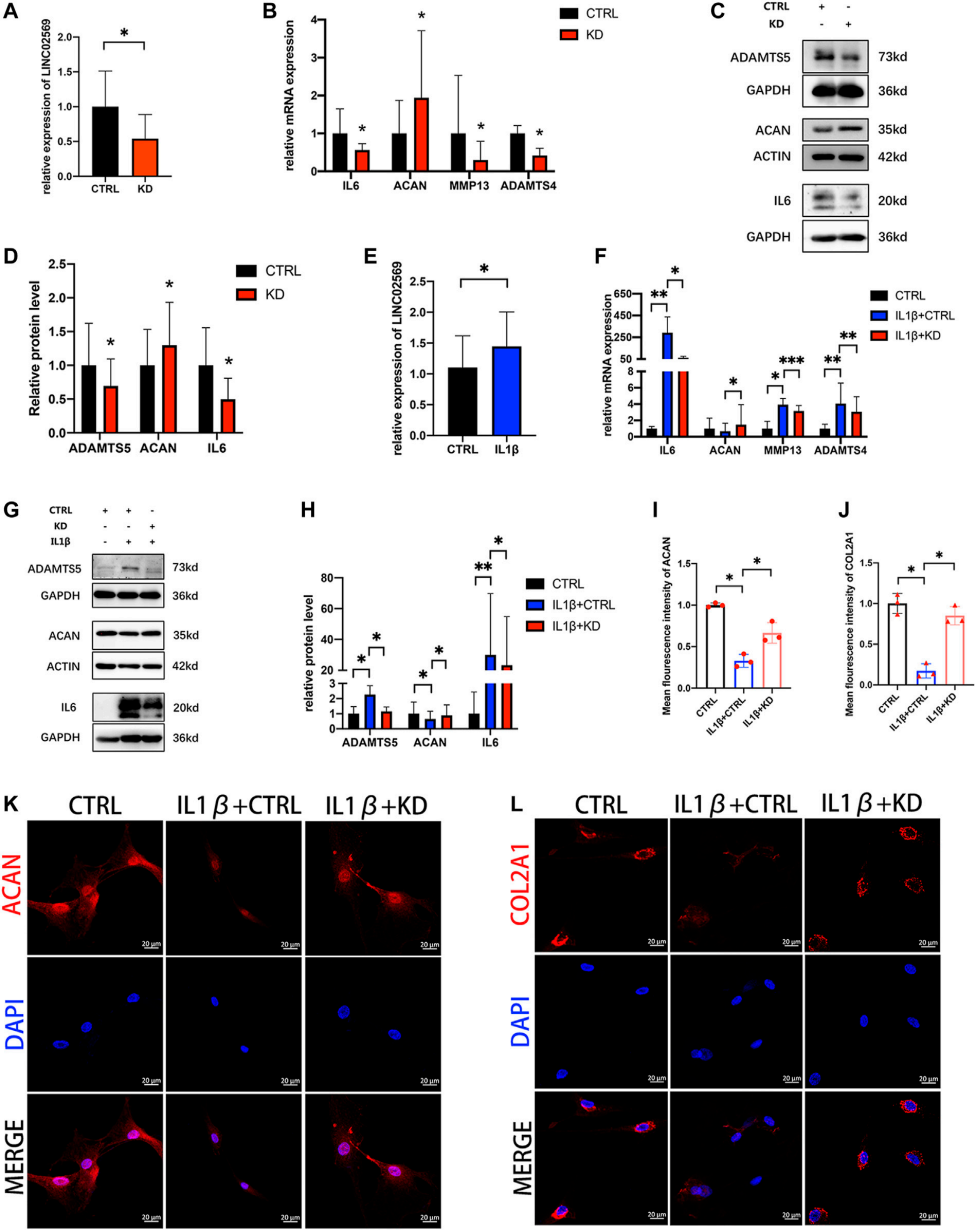
Knockdown of LINC02569 attenuated NPCs degeneration. **(A)** qRT-PCR assay of LINC02569 expression in LINC02569 knockdown NPCs. **(B)** qPCR assay of IL6, ACAN, MMP13, ADAMTS4 expression in LINC02569 knockdown NPCs. **(C,D)** Protein levels of IL6, ACAN and ADAMTS5 determined by WB analyses of lysates from LINC02569 knockdown NPCs. **(E)** qRT-PCR assay of LINC02569 expression in NPCs treated with IL-1β for 24 h. **(F)** qRT-PCR assay of IL6, ACAN, MMP13 and ADAMTS4 expression in LINC02569 knockdown NPCs treated with IL-1β. **(G,H)** Protein levels of IL6, ACAN and ADAMTS5 determined by WB analyses of lysates from LINC02569 knockdown NPCs treated with IL-1β. (K, L) Representative confocal images of ACAN (K) and COL2A1 (L) formation. Scale bar: 20 μm. **(I)** Quantitation of panel **(K)**. **(J)** Quantitation of panel **(L)**. Quantitative results are from 3 independent experiments and are shown as the mean ± SD. ∗*p* < 0.05, ∗∗*p* < 0.01, ***p < 0.001.

To investigate whether LINC02569 expression was associated with NPCs degeneration, knockdown of LINC02569 was performed (Figure 4A). Inflammation related gene (IL6) and ECM degeneration related genes (ACAN, MMP13, ADAMTS4 and ADAMTS5) was investigated. The results showed that reduction of LINC02569 expression led to mRNA level reduction of IL6, MMP13, ADAMTS4 and increase of ACAN (Figure 4B), meanwhile led to protein level reduction of IL6, ADAMTS5 and increase of ACAN (Figures 4C,D). LINC02569 could be up-regulated by IL-1β stimulation (Figure 4E), and knockdown of LINC02569 reversed IL-1β-induced inflammation and ECM degeneration (Figures 4F–L).

**FIGURE 5:**
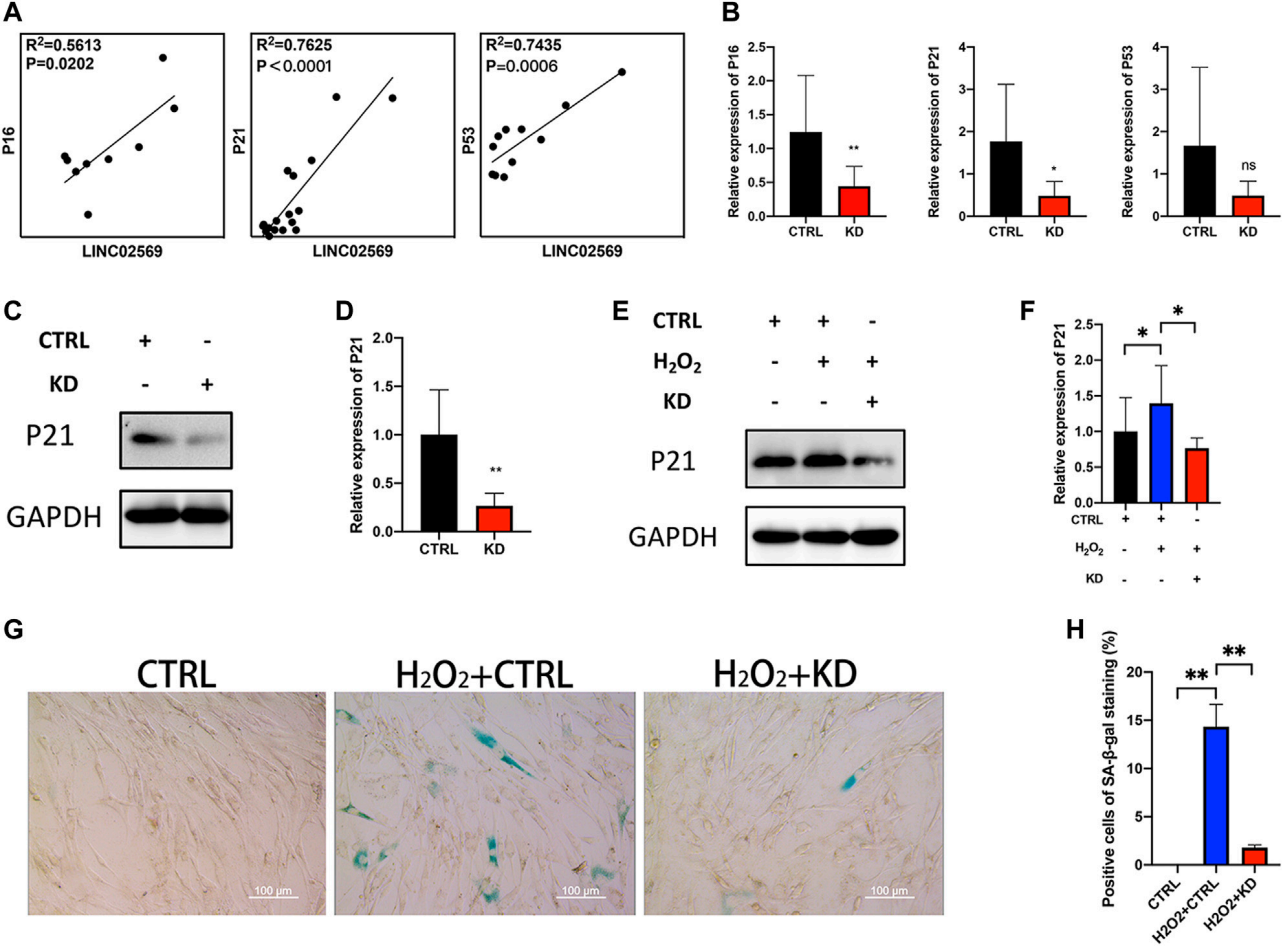
Knockdown of LINC02569 alleviated NPCs senescence. **(A)** Scatter plot of qRT-PCR assay showed correlation of expression between LINC02569 and P16, P21 and P53. *R* ^2^ and *p* values were determined by Pearson’s correlation test. **(B)** qRT-PCR assay of P16, P21 and P53 expression in LINC02569 knockdown NPCs. **(C,D)** Protein levels of P21 determined by WB analyses of lysates from LINC02569 knockdown NPCs. **(E,F)** Protein levels of P21 determined by WB analyses of lysates from LINC02569 knockdown NPCs treated with H_2_O_2_. **(G,H)** Representative SA-β-gal staining of NPCs from different groups. Scale bar: 100 μm. Quantitative results are from 3 independent experiments and are shown as the mean ± SD. ∗ *p* < 0.05, ∗∗ *p* < 0.01.

Since cell senescence was reported to be closely related to NP degeneration (Novais et al., 2019; Che et al., 2020; Cherif et al., 2020), senescence related genes (P16, P21 and P53) were investigated. qRT-PCT assay of NP tissue isolated RNA showed positive correlation between LINC02569 and senescence related genes (Figure 5A), suggesting that LINC02569 expression was correlated with NP senescence. Knockdown of LINC02569 reduced mRNA level of P16 and P21 (Figure 5B) and protein level of P21 (Figures 5C,D), meanwhile reversed H_2_O_2_-induced NPCs senescence (Figures 5E–H).

### Knockdown of LINC02569 Attenuated Nucleus Pulposus Cells Degeneration by Blocking NF-κB Signaling Pathway

**FIGURE 6:**
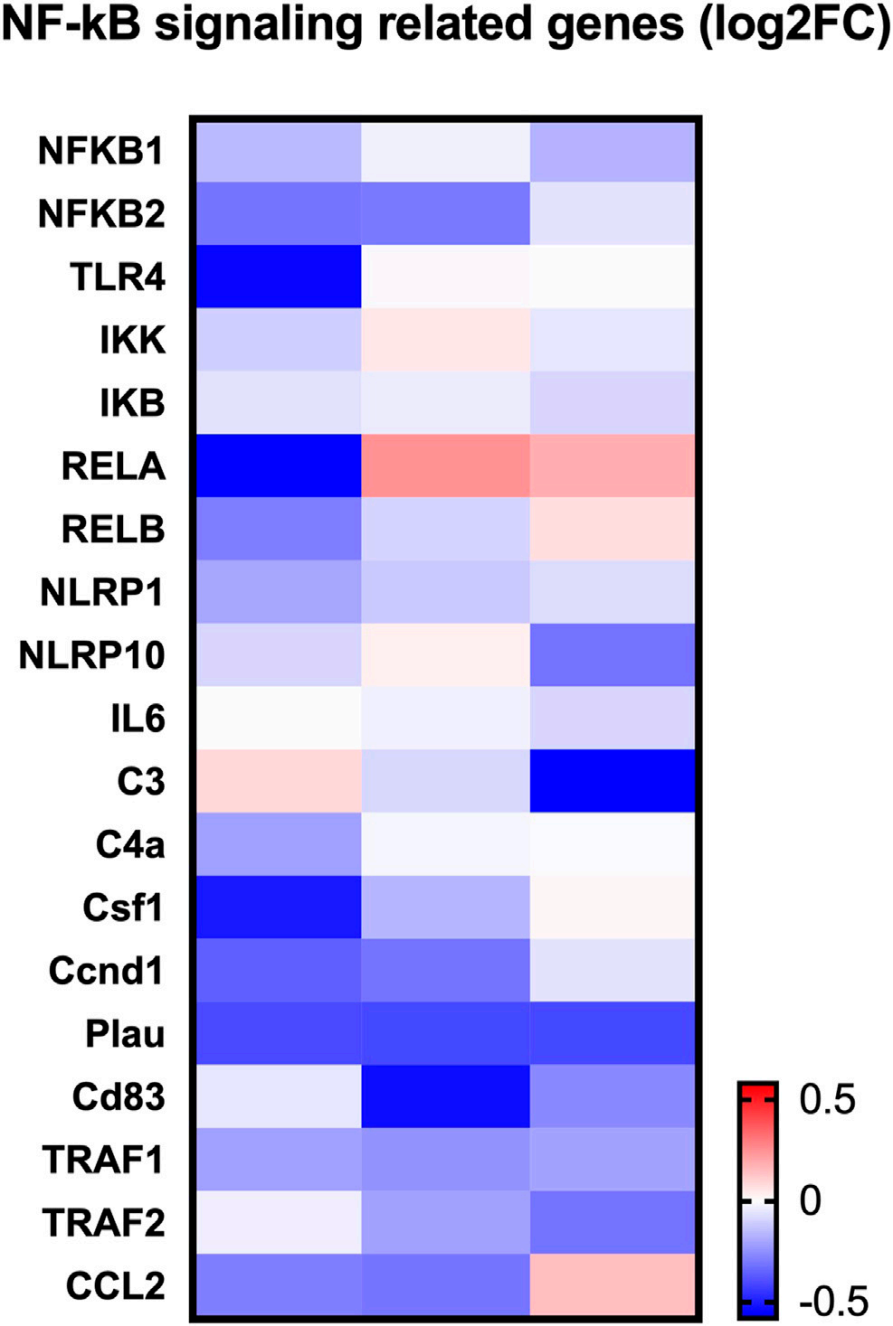
Heatmap showed expression change of NF-κB related genes between LINC02569 knockdown group and negative control group in NPCs from RNA-seq.

To determine how LINC02569 play role in the regulation of NP degeneration, RNA-seq was performed on normal control and LINC02569 knockdown NPCs. Key genes and target genes of NF-κB signaling pathway were found to be down-regulated (Figure 6).

**FIGURE 7:**
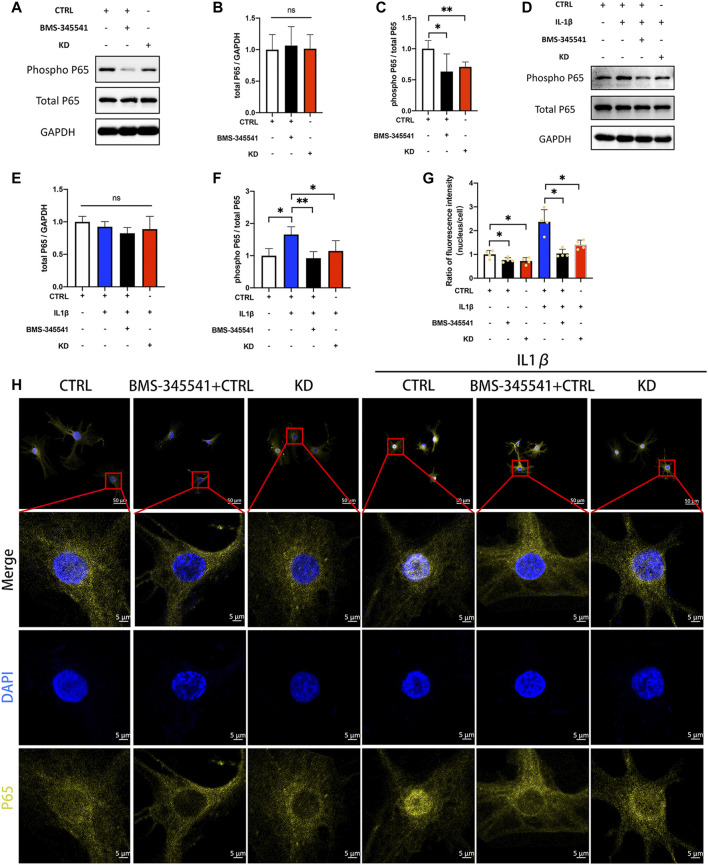
Knockdown of LINC02569 attenuated NPCs degeneration by blocking NF-κB signaling pathway. **(A–C)** Protein levels of total P65 and phosphorylated P65 determined by WB analyses of lysates from LINC02569 knockdown NPCs or NPCs treated with BMS-345541 (NF-κB inhibitor). **(D–F)** Protein levels of total P65 and phosphorylated P65 determined by WB analyses of lysates from LINC02569 knockdown NPCs or NPCs treated with BMS-345541 and IL-1β. **(G–H)** Representative immunofluorescence images of total P65 in NPCs from different groups. Quantitative results are from 3 independent experiments and are shown as the mean ± SD. ∗ *p* < 0.05, ∗∗ *p* < 0.01.

We next determined whether suppression of LINC02569 attenuated NPCs degeneration by blocking NF-κB signaling pathway. IL-1β at 5 ng/ml/BMS-345541 at 20uM were proved to be the appropriate concentration to activate/suppress NFκB pathway in NPCs (Supplementary Figure 1A–G). WB analysis confirmed that the ratio of phosphorylated P65/total P65 was down-regulated in LINC02569 knockdown NPCs (Figures 7A,C, *p* < 0.01), while total P65 showed no significant difference (Figures 7A,B). Similarly, the knockdown also restored the activation of P65 phosphorylation by IL-1β (Figures 7D,F, *p* < 0.05), while total P65 showed no significant difference (Figures 7D,E). Besides, immunofluorescence analysis confirmed that suppression of LINC02569 decreased nucleus translocation of P65 and restored increased nucleus translocation of P65 induced by IL-1βin NPCs (Figures 7G,H, *p* < 0.05).

## Discussion

**FIGURE 8:**
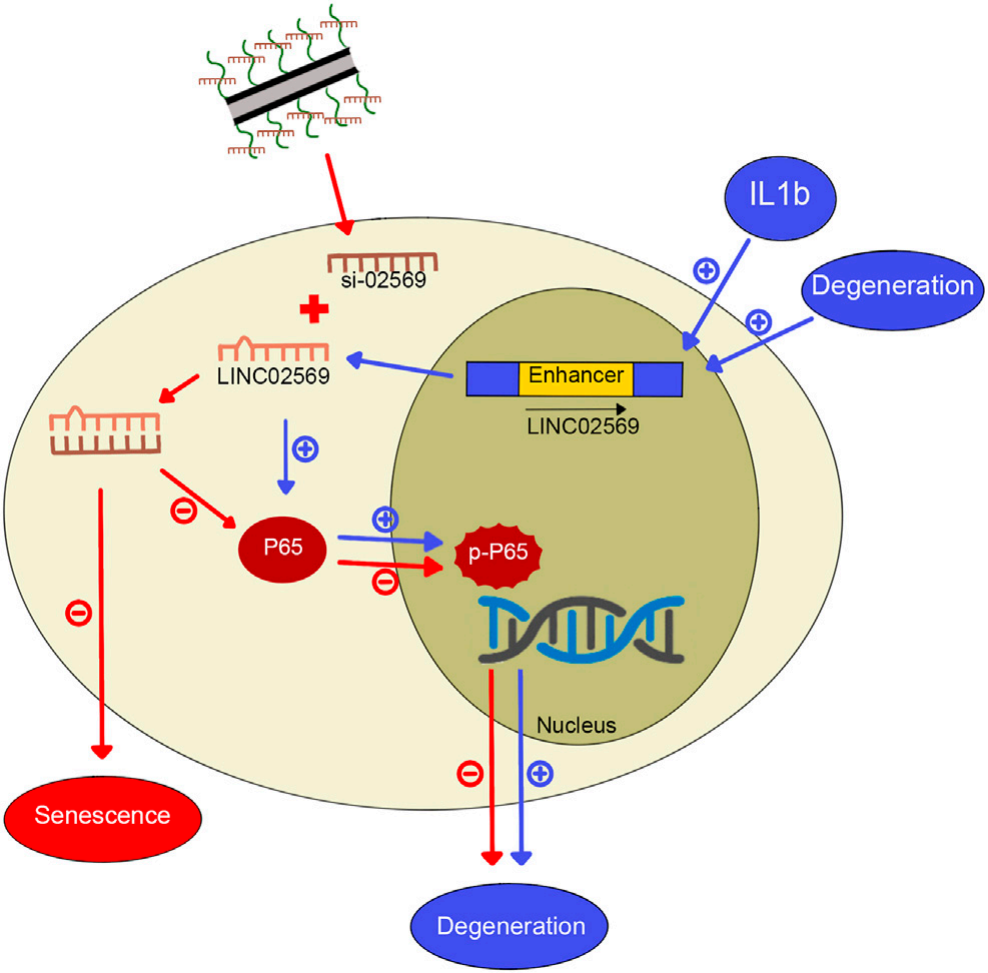
Graphical abstract for IL1β-induced LINC02569 promoted NPC degeneration through NF-κB. 1) LINC02569 was upregulated in degenerative NP or IL-1β-stimulated NPCs. LINC02569 promoted NF-kB activation, leading to the degeneration of NPCs. (See pathway consist of blue arrows. “+” represents promoting). 2) Silencing LINC02569 with oCNT-pb-meditated siRNA inhibited P65 phosphorylation and prevented the transfer into the nucleus, resulting in attenuation of NP degeneration. Moreover, LINC02569 silencing leaded to alleviation of cell senescence. (See pathway consist of red arrows. “−” represents restraining).

Intervertebral disc (IVD) is a complex fibrocartilaginous tissue that connects adjacent vertebral bodies and maintain mechanical loading to enable spinal motion. IDD, which is a widely recognized cause of back pain (Smith et al., 2011), worsens with age, and more than 80% of IVDs exhibit degeneration-related changes in people over 50 years old (Vo et al., 2016). The widely used treatment strategy for IDD consists of physiotherapy, drug therapy and surgery. This approach, however, has shown limited success. Recently, gene therapy seems to be a promising approach to delay or even reverse IDD, whereas the delivery system used to transfer exogenous genes into IVD cells remains a challenge (Blanquer et al., 2015; Micheletti et al., 2017). In this study, we identified a key lncRNA (LINC02569) with the Microarray, and demonstrated that using our previously designed cationic polymer brush-modified CNT-based siRNA delivery platform (Li et al., 2021) to transport si-02569 to NPCs, not only significantly increased the transfection efficiency but also decreased the potential toxicity. In addition, delivery of si-02569 with this nanoparticle platform could significantly alleviate the inflammatory response in the IL1β-induced degenerated NPCs via regulating NF-κB signaling pathway (Figure 8). These results provide us a promising alternative potential therapeutic strategy for biological intervention of IDD.

LncRNAs have been confirmed to play crucial roles in the regulation of various diseases (He et al., 2014; Zhu et al., 2019; Lin et al., 2020) and biological processes, including cellular differentiation, proliferation, apoptosis and gene regulation (Klattenhoff et al., 2013; Chen J. et al., 2017; Zhu et al., 2019). Thus, they have attracted more and more significant attention because of their imperative roles. For example, Huang et al. used microarray to obtain the expression profiles of lncRNAs in osteoarthritis (OA) cartilage and predicted the potential function and targets, which indicated them as the new biomarkers for diagnosis or novel therapeutic targets of OA (Fu et al., 2015). The elncRNAs are annotated lncRNAs in which initiation sites overlap with ehancer regions. Compared to other types of eRNAs, elncRNAs are acknowledged more stable (Li et al., 2016). Moreover, the interaction frequency of elncRNA-associated enhancer-promoter pairs was significantly higher than that of other enhancer-promoter pairs, suggesting that elncRNAs may regulate the gene expression more powerfully (Hou et al., 2019). Although, elncRNAs have been proved to play crucial roles in some human disease (Chowdhury et al., 2018), whether these kind of lncRNAs regulate IDD initiation and progression remains largely unknown. In this study, with the use of Microarray, we examined the elncRNA expression profiles of degenerated NP tissues, and identified LINC02569 as a key factor in regulating IDD. After using dozens of clinical samples to validate the association of LINC02569 with degenerative grades and exploring its molecular mechanism based on the RNA sequences, we demonstrated that this elncRNA can augment inflammatory response of NPCs via activating the NF-κB signaling pathway, which could be used as a potential therapeutic target for suppressing inflammation of IDD.

The inflammatory response and subsequent inflammatory-associated factors IL-1 β, IL-6 and TNF-α have been widely presented to be associated with IDD (Zhang et al., 2019). It has been reported that IL-1β and TNF-α are highly expressed in degenerative IVD tissues (Wei et al., 2015; Wei et al., 2019; Wang et al., 2020; Zhang et al., 2020). These cytokines, expressed by NPCs, are involved in multiple pathological progresses, including matrix degradation, cellular apoptosis, senescence and oxidative stress (Hoyland et al., 2008; Yang et al., 2015). Matrix Metallo Proteinases (MMP) and A Disintegrin And Metalloproteinases with Thrombospondin Motifs proteins (ADAMTS) are 2 important families of enzymes to mediate matrix degradation (Vo et al., 2013). Although many studies indicate that the expression levels of MMP and ADAMTS are regulated by a combination of many factors, including mechanical, inflammatory, and oxidative stress, some of which are mediated in part through the p38 mitogen-activated protein kinase and NF-κB signaling pathway. Inflammation is thought to be the main factor. The matrix degradation products then alleviated MMP inhibition and trigger inflammation in return, implying a cascade of structurally disrupting events (Marom et al., 2007). In our study, we also found that both MMP-13, ADAMTS4 and ADAMTS-5 were highly upregulated in the degenerated NPCs, which were in line with the inflammatory-associated factor IL-1β. This was consistent with the previous studies (Molinos et al., 2015). IL-1β stimulation has been widely used to establish NPC degeneration model (Lu et al., 2019; Wang et al., 2019). In this study, we also used IL-1β to treat NPCs for 24 h and found that the concentration of 5 ng/ml could sufficiently trigger the initiation of NPCs degeneration, which was lower than previous report (Wang et al., 2019). This might be due to the status of the cells individually. Various intracellular signaling pathways are activated in response to inflammatory stimulation during the process of IDD, subsequently mediating the increase in the secretion of a downstream effector that is closely involved in the progress of IDD (Wuertz et al., 2012). Being considered as one of the most important intracellular signaling proteins, NF-κB plays a crucial role in regulating the expression of genes associated with extracellular matrix (ECM) degradation in IL-1β-treated human NPCs (Feng et al., 2016; Kang et al., 2017). Suppressing the activation of NF-κB has been regarded as a potential therapeutic strategy against IDD. IκB, which is an inhibitory protein bonding NF-κB in the cytoplasm, prevents NF-κB from entering the nucleus under normal conditions. When stimulated by Il-1β, the IκB protein is phosphorylated and degraded, resulting in NF-κB phosphorylation and translation of NF-κB from the cytoplasm to the nucleus, subsequently upregulating the secrection of catabolic enzymes, inflammatory mediators and cytokines (Johnson et al., 2015; Chen Y. G. et al., 2017). In our study, to further elucidate the molecular mechanism underlying the effects of LINC02569 on IDD, we performed the RNA sequence analysis for the NPCs with and without LINC02569 knockdown, and found that the key genes and target genes of NF-κB pathway were downregulated, which suggested the potential association between LINC02569 and NF-κB signaling pathway. After delivery of si-02569 with oCNT-pb, the results revealed that the phosphorylation of P65, along with the nucleus translocation of P65, was significantly suppressed in NPCs with or without stimulation of IL1β. Subsequently, the degeneration of NPCs was alleviated confirmed by the expression of aggrecan and collagen II.

On the other hand, cellular senescence was reported to be a contributor to IDD and LBP (Cherif et al., 2020). In IDD, cellular senescence accumulates and is associated with reduced proliferation and increased inflammatory response. Senescence may occur as part of natural aging process, but can also be accelerated by growth factor deficiency, oxidative accumulation, and inflammatory stimulation (Wang et al., 2016). Reduction of senescent cells and senescence-associated secretory phenotype (SASP) could result in ECM improvement (Cherif et al., 2020). We demonstrated that suppression of LINC02569 decreased the expression of SASP, such as P16, P21 and P53, resulting in the alleviation of senescence. The results above suggest that elncRNA LINC02569 might be a promising therapeutic target in suppression of inflammation induced NP degeneration and senescence.

Accompanied by extensively elucidating numerous functional lncRNAs and their regulatory mechanisms, efforts to improve synthetic gene delivery have led to the development of various delivery systems (Allen and Cullis, 2013; You et al., 2018; Bi et al., 2020; Zhou et al., 2020; Zhang et al., 2021). RNA interference (RNAi) technology is widely used due to its remarkable ability to silence the expression of target genes. Due to the polyanionic and biomacromolecular characters of RNAi technique, various delivery vehicles have been developed to improve siRNA delivery (Ali et al., 2017; Xu et al., 2018). Previous studies have reported that nanoparticles showed great promise for improvement of siRNA delivery, and surprisingly, some of the nanoparticle platforms have been marketed or projected into the clinical trials to treat human diseases (Shi et al., 2017). Recently, carbon nanotubes (CNTs), which possess unique characters (i.e., hollow structure, high aspect ratios) have made themselves more attractive (Krishnamoorthy et al., 2017). However, efficient and safe systemic delivery of siRNAs into NPCs remains challenging. Cationic polymer brushes synthesized via surface-initiated atom transfer radical polymerization (SI-ATRP) have attracted more attention for the design of siRNA delivery systems, as they offer flexibility for tailoring the surface chemistries and architectures (Li et al., 2021). It has been confirmed that the cationic polymer brushes could significantly alter the surface properties of CNTs, such as hydrophilicity, biofunctionality (Zhang et al., 2017). In this study, we used our previously designed CNTs, which were modified and functionalized by the cationic polymer brushes, to deliver si-02569 to NPCs. The results demonstrated that it showed lower toxicity accompanied by higher transfection efficiency, compared with the traditional siRNA delivery system. These results inferred that the high hydrophilicity induced by cationic polymer brush modification and structural adjustment could increase the cellular uptake of siRNA with low toxicity. Taken together, these results highlight the potential for CNTs-based siRNA delivery of elncRNA as a therapeutic strategy for the amelioration of inflammation mediated IDD.

Several limitations of this study should be acknowledged. First, we did not perform the *in vivo* study to evaluate the efficiency of siRNA delivery. Animal study is essentially imperative for preparation of clinical translation. In addition, although the effects of elncRNA LINC02569 on inflammation-related IDD have been verified, the deep molecular mechanisms at the downstream was not studied, due to the limited funding. Hence, further studies are warranted in the near future to overcome the limitations mentioned above.

In conclusion, our results demonstrated that delivery of si-02569 with oCNT-pb platform could significantly alleviate the inflammation of degenerative NPCs via suppressing NF-κB signaling pathway, besides, alleviated the senescence of NPCs. This result highlights the potential for oCNT-pb-based siRNA delivery of elncRNA as a therapeutic strategy for the amelioration of inflammation mediated IDD.

## Data Availability

The datasets presented in this study can be found in online repositories. The names of the repository/repositories and accession number(s) can be found below: https://www.ncbi.nlm.nih.gov/geo/, GSE185668 https://www.ncbi.nlm.nih.gov/geo/, GSE185876.
