# Supplementary material for: Cationic Polymer Brush-Modified Carbon Nanotube-Meditated eRNA LINC02569 Silencing Attenuates Nucleus Pulposus Degeneration by Blocking NF-κB Signaling Pathway and Alleviate Cell Senescence
Source: Front Cell Dev Biol. 2022 Jan 17;9:837777. doi: 10.3389/fcell.2021.837777 (PMC8802762; doi:10.3389/fcell.2021.837777)
Supplement: Supplementary file 1 [file Presentation1.PPTX]

## Slide 1
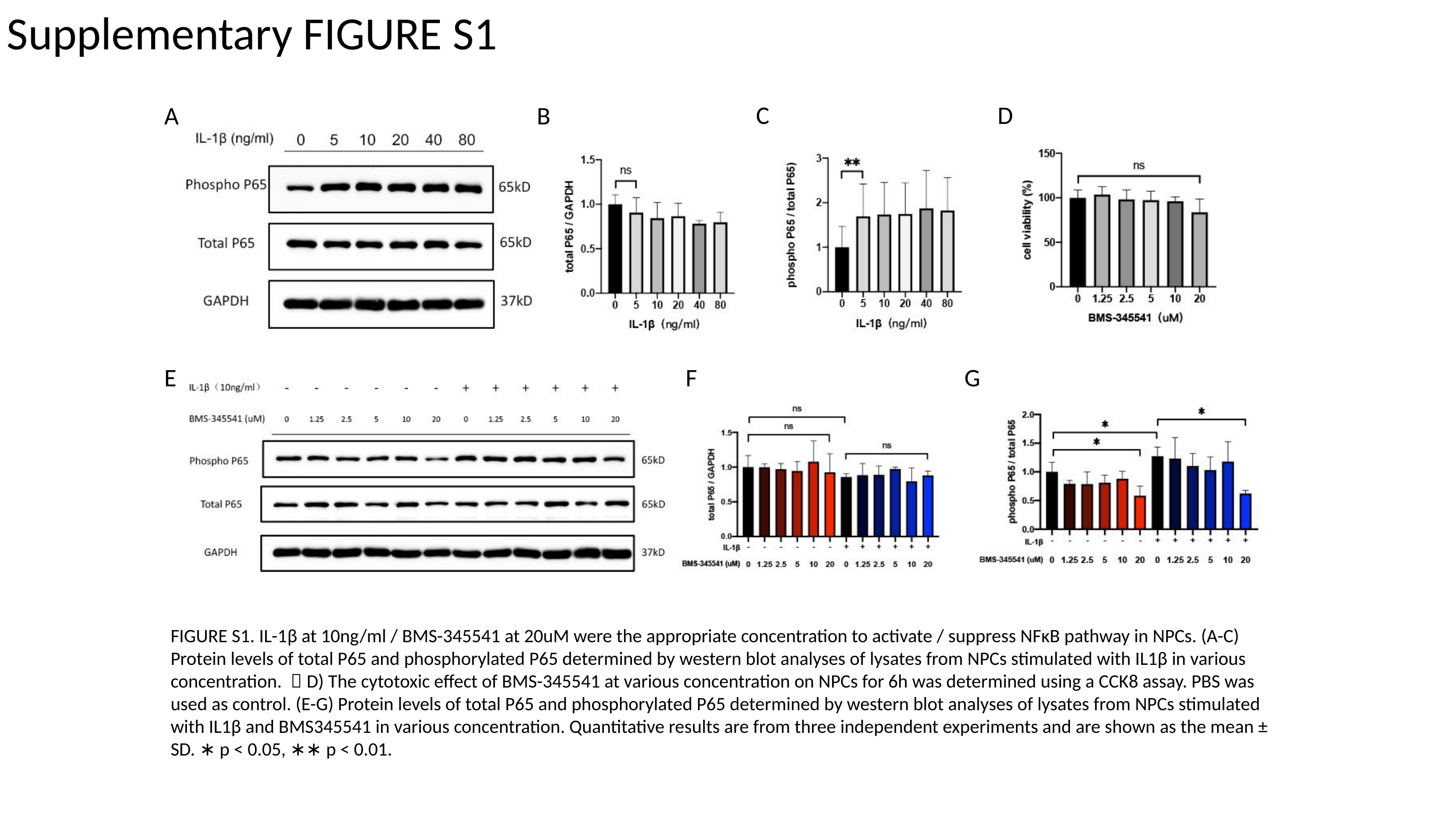

Supplementary FIGURE S1
C
D
A
B
F
G
E
FIGURE S1. IL-1β at 10ng/ml / BMS-345541 at 20uM were the appropriate concentration to activate / suppress NFκB pathway in NPCs. (A-C) Protein levels of total P65 and phosphorylated P65 determined by western blot analyses of lysates from NPCs stimulated with IL1β in various concentration. （D) The cytotoxic effect of BMS-345541 at various concentration on NPCs for 6h was determined using a CCK8 assay. PBS was used as control. (E-G) Protein levels of total P65 and phosphorylated P65 determined by western blot analyses of lysates from NPCs stimulated with IL1β and BMS345541 in various concentration. Quantitative results are from three independent experiments and are shown as the mean ± SD. ∗ p < 0.05, ∗∗ p < 0.01.
