# Supplementary material for: Cationic Polymer Brush-Modified Carbon Nanotube-Meditated eRNA LINC02569 Silencing Attenuates Nucleus Pulposus Degeneration by Blocking NF-κB Signaling Pathway and Alleviate Cell Senescence
Source: Front Cell Dev Biol. 2022 Jan 17;9:837777. doi: 10.3389/fcell.2021.837777 (PMC8802762; doi:10.3389/fcell.2021.837777)
Supplement: Supplementary file 2 [file Presentation3.PPTX]

## Slide 1
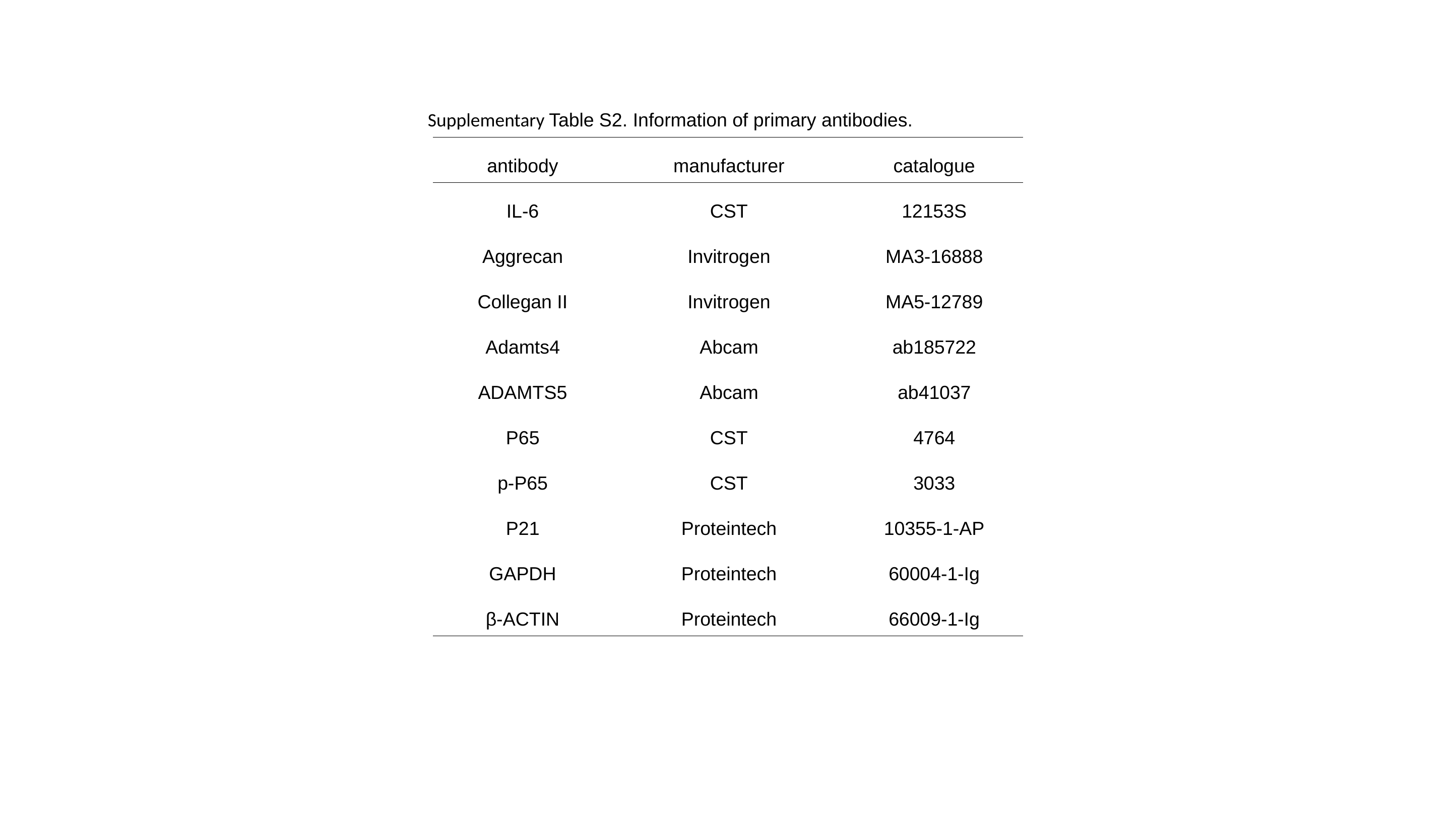

Supplementary Table S2. Information of primary antibodies.
| antibody | manufacturer | catalogue |
| --- | --- | --- |
| IL-6 | CST | 12153S |
| Aggrecan | Invitrogen | MA3-16888 |
| Collegan II | Invitrogen | MA5-12789 |
| Adamts4 | Abcam | ab185722 |
| ADAMTS5 | Abcam | ab41037 |
| P65 | CST | 4764 |
| p-P65 | CST | 3033 |
| P21 | Proteintech | 10355-1-AP |
| GAPDH | Proteintech | 60004-1-Ig |
| β-ACTIN | Proteintech | 66009-1-Ig |
