# Supplementary material for: Cationic Polymer Brush-Modified Carbon Nanotube-Meditated eRNA LINC02569 Silencing Attenuates Nucleus Pulposus Degeneration by Blocking NF-κB Signaling Pathway and Alleviate Cell Senescence
Source: Front Cell Dev Biol. 2022 Jan 17;9:837777. doi: 10.3389/fcell.2021.837777 (PMC8802762; doi:10.3389/fcell.2021.837777)
Supplement: Supplementary file 3 [file Presentation2.PPTX]

## Slide 1
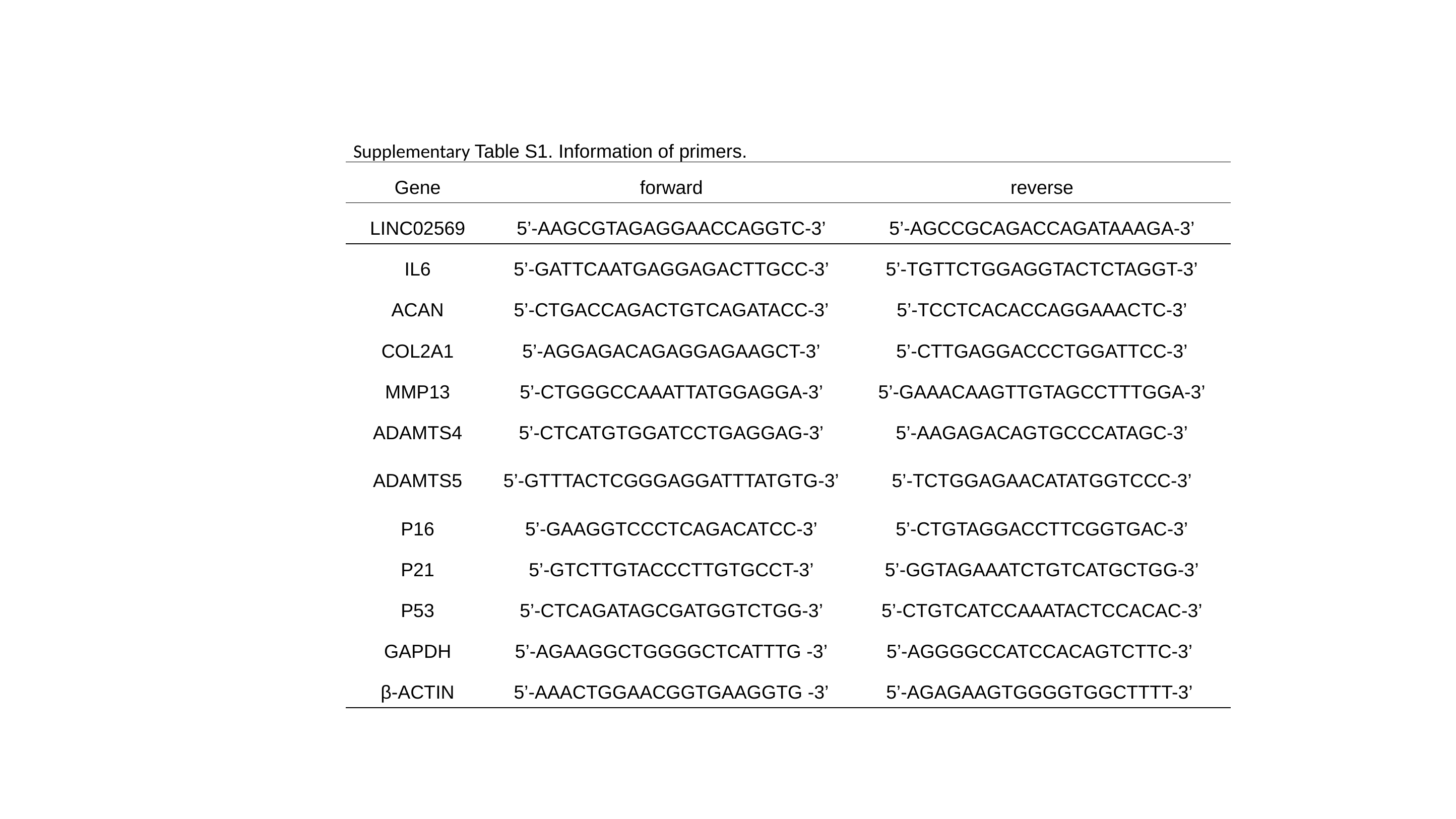

Supplementary Table S1. Information of primers.
| Gene | forward | reverse |
| --- | --- | --- |
| LINC02569 | 5’-AAGCGTAGAGGAACCAGGTC-3’ | 5’-AGCCGCAGACCAGATAAAGA-3’ |
| IL6 | 5’-GATTCAATGAGGAGACTTGCC-3’ | 5’-TGTTCTGGAGGTACTCTAGGT-3’ |
| ACAN | 5’-CTGACCAGACTGTCAGATACC-3’ | 5’-TCCTCACACCAGGAAACTC-3’ |
| COL2A1 | 5’-AGGAGACAGAGGAGAAGCT-3’ | 5’-CTTGAGGACCCTGGATTCC-3’ |
| MMP13 | 5’-CTGGGCCAAATTATGGAGGA-3’ | 5’-GAAACAAGTTGTAGCCTTTGGA-3’ |
| ADAMTS4 | 5’-CTCATGTGGATCCTGAGGAG-3’ | 5’-AAGAGACAGTGCCCATAGC-3’ |
| ADAMTS5 | 5’-GTTTACTCGGGAGGATTTATGTG-3’ | 5’-TCTGGAGAACATATGGTCCC-3’ |
| P16 | 5’-GAAGGTCCCTCAGACATCC-3’ | 5’-CTGTAGGACCTTCGGTGAC-3’ |
| P21 | 5’-GTCTTGTACCCTTGTGCCT-3’ | 5’-GGTAGAAATCTGTCATGCTGG-3’ |
| P53 | 5’-CTCAGATAGCGATGGTCTGG-3’ | 5’-CTGTCATCCAAATACTCCACAC-3’ |
| GAPDH | 5’-AGAAGGCTGGGGCTCATTTG -3’ | 5’-AGGGGCCATCCACAGTCTTC-3’ |
| β-ACTIN | 5’-AAACTGGAACGGTGAAGGTG -3’ | 5’-AGAGAAGTGGGGTGGCTTTT-3’ |
